# First-in-human pilot trial of G-CSF–mobilised platelet–leukocyte concentrate for severe refractory Asherman syndrome

**DOI:** 10.3389/fendo.2026.1923604

**Published:** 2026-08-13

**Authors:** BingJie Rui, YanFeng Yang, Hye Kyung Yoon, Jae Eun Jung, Ji Hyang Kim

**Affiliations:** 1 Department of Obstetrics and Gynecology, CHA University School of Medicine, Pocheon, Republic of Korea; 2 Fertility Center, CHA University Bundang Medical Center, Seongnam, Republic of Korea; 3 Department of Obstetrics and Gynecology, Fushun Second Hospital, Fushun, China; 4 Department of Obstetrics and Gynecology, Fushun Maternal and Child Health Hospital, Fushun, China; 5 Department of Obstetrics and Gynecology, Infertility Center, CHA University Ilsan Medical Center, Goyang, Republic of Korea

**Keywords:** endometrial regeneration, G-CSF, infertility, peripheral blood platelet–leukocyte concentrate, severe refractory Asherman syndrome

## Abstract

**Introduction:**

Severe refractory Asherman syndrome (SRAS) remains difficult to treat, and the benefit of regenerative blood-derived therapies remains uncertain. This study evaluated whether systemic granulocyte colony-stimulating factor (G-CSF) mobilisation combined with targeted intrauterine delivery of autologous peripheral blood platelet–leukocyte concentrate (PBPLC) could improve endometrial thickness in women with SRAS.

**Methods:**

This prospective, single-centre, single-arm pilot study enrolled 10 women with infertility for at least 3 years, severe intrauterine adhesions, poor endometrial development, and failure of previous adhesiolysis, hormonal therapy, and platelet-rich plasma treatment. After G-CSF mobilisation, autologous PBPLC was injected into the endometrial–myometrial junction under hysteroscopic guidance without concurrent formal adhesiolysis. The primary outcome was the change in late pre-ovulatory endometrial thickness (LPO-EMT), analysed using the exact Wilcoxon signed-rank test and Hodges–Lehmann estimates.

**Results:**

LPO-EMT increased from 4.6 mm (SD 0.7) to 5.8 mm (SD 1.3), with a Hodges–Lehmann paired difference of 1.08 mm (95% CI 0.45–2.50; p = 0.0039). Hysteroscopic cavity restoration, reduced adhesion severity, and subjective menstrual improvement were observed in all participants, and the AFS score decreased from 10.8 to 4.5. No grade 3 or higher treatment-related adverse events occurred. Among nine women who attempted conception, pregnancy was detected in six (66.7%; exact 95% CI 29.9–92.5), including four clinical pregnancies (44.4%; exact 95% CI 13.7–78.8). However, all clinical pregnancies ended in first-trimester miscarriage, and no ongoing pregnancies or live births were achieved during follow-up.

**Discussion:**

These preliminary findings suggest that G-CSF-mobilised autologous PBPLC may be a feasible approach for improving endometrial thickness and uterine cavity restoration in women with SRAS. However, the findings do not establish reproductive efficacy, and larger controlled studies are warranted.

**Clinical trial registration:**

## 1 Introduction

Asherman syndrome (AS), characterized by intrauterine adhesions (IUAs) and partial or complete obliteration of the uterine cavity, is a devastating cause of menstrual disturbance and infertility (1). The severity of IUAs in AS is commonly assessed using classification systems such as those proposed by the AFS and the European Society for Gynaecological Endoscopy (ESGE). The AFS system categorizes adhesions as mild (1–4 scores), moderate (5–8 scores), or severe (9–12 scores) on the basis of type of adhesions, menstrual history, and the extent of uterine cavity involvement (2). The ESGE system grades adhesions primarily according to hysteroscopic findings, including adhesion type, location, and extent (3). Diagnosis and staging are typically established during diagnostic hysteroscopy. Recurrence rates as high as 63% have been reported in severe AS, which is associated with reduced surgical success and poorer fertility outcomes (4). These factors collectively reduce the likelihood of endometrial regeneration and of conceiving and carrying a pregnancy to term.

Current management strategies for SRAS focus on mechanical restoration of the uterine cavity and prevention of re-adhesion, including repeat adhesiolysis, solid/semi-solid barriers, and postoperative estrogen-based hormone therapy (3). Despite these measures, outcomes remain suboptimal when the basalis is extensively compromised. In this setting, failure of endometrial regeneration is increasingly viewed as a biological problem—depletion of endogenous reparative cells coupled with a hostile microenvironment—rather than a purely anatomic problem. Recent translational studies suggest that the post-injury niche in SRAS is characterized by persistent inflammation, aberrant remodeling, and pro-fibrotic signaling, all of which may limit restoration of a receptive lining even after technically successful adhesiolysis (5, 6). In such “end-stage” uteri, regenerative approaches may be needed not only to reopen the cavity and restore the endometrial surface, but also to address persistent dysfunction of deeper uterine compartments — including the basalis, junctional zone, and subendometrial vasculature — that may not recover with adhesiolysis-based or endometrium-centric strategies alone.

Among emerging approaches, platelet-rich plasma (PRP) provides a concentrated mixture of growth factors and cytokines with potential pro-angiogenic, pro-remodeling, and immunomodulatory effects (7). Our prior research has shown that PRP significantly promotes implantation rate and live birth rate in patients with refractory thin endometrium and implantation failures (8). In parallel, G-CSF is the most commonly used agent to mobilize peripheral blood hematopoietic stem and progenitor cells for collection and hematopoietic cell transplantation (9). Beyond its role in mobilization, G-CSF is also a therapy for congenital and acquired neutropenias because of its potent stimulation of neutrophil production (10). Although neutrophils recruited during acute inflammation can exacerbate tissue injury through the release of reactive oxygen species, proteolytic enzymes, and pro-apoptotic mediators, accumulating evidence indicates that the innate immune response is not purely destructive. During the resolution phase, functionally distinct neutrophil subsets can emerge that support tissue repair and re-establishment of homeostasis (11, 12). Importantly, G-CSF administration can alter the cellular composition and immunophenotype of circulating mononuclear leukocytes, thereby potentially influencing inflammatory resolution and tissue repair programs (13). Taken together, PBPLC to the injured basalis may provide both a cellular “seed” and a growth factor–rich milieu in a minimally invasive, fully autologous manner.

We therefore conducted a prospective, single-arm trial to investigate the feasibility, safety, and preliminary efficacy of hysteroscopic intra-endometrial injection of PBPLC after G-CSF mobilisation in women with SRAS. Anticipating that, in this end-stage population, the disease process may extend beyond the endometrial surface to involve the basalis, the endometrial–myometrial junction, and subendometrial tissue, we deliberately designed a dual-axis regenerative regimen rather than a surface intrauterine infusion. The regimen combined (i) a systemic component — G-CSF mobilisation to recruit circulating leukocytes and a small population of haematopoietic progenitor cells into the peripheral blood-derived product — with (ii) a deep local component — hysteroscopic subendometrial injection of this product into multiple sites at the endometrial–myometrial junction. We hypothesised that this combined local–systemic strategy would increase endometrial thickness, improve hysteroscopic appearance of the uterine cavity, and generate pregnancy signals in this extremely poor-prognosis population, while simultaneously providing a stringent test of whether intrauterine regenerative therapy — even when augmented with systemic mobilisation and deep subendometrial delivery — is sufficient to overcome the reproductive barrier of severe refractory disease. The findings are intended to inform the benefit–risk ratio of this strategy and, by examining the relationship between anatomical and endometrial recovery on the one hand and reproductive outcomes on the other, to help clarify whether severe refractory Asherman syndrome is adequately captured by an endometrium-centric disease model or whether it should instead be conceptualised as a whole-uterus disorder requiring compartment-spanning regenerative approaches in future trials.

## 2 Materials and methods

### 2.1 Study design

This was a single-center, prospective, single-arm trial conducted at CHA University Fertility Center Bundang (Gyeonggi-do, South Korea) between 11 June 2019 and 1 July 2022. The protocol was approved by the Ethics Committee of CHA University Fertility Center Bundang (IRB number: CHAMC 2019-03-037-005), and written informed consent was obtained from all participants. This registered platform trial (KCT0004208) evaluated feasibility, safety, and preliminary clinical activity, including changes in endometrial thickness and exploratory reproductive outcomesin women with SRAS. The trial was registered on 5 June 2019, and the first participant was enrolled on 11 June 2019.

### 2.2 Study population

Eligible participants were women aged 20–44 years with normal ovarian reserve, BMI <30 kg/m^2^, and investigator-assessed ovulatory status. They had SRAS with infertility lasting ≥3 years, persistent thin endometrium, intrauterine adhesions despite prior treatment, and at least one previous failed IVF cycle. Severe Asherman syndrome was defined according to the AFS intrauterine adhesion scoring system, with the extent of cavity involvement and adhesion type assessed by hysteroscopy and pelvic ultrasonography. Refractory disease was defined as persistent oligo/amenorrhea despite completing at least two cycles of high-dose estrogen–progestin therapy after adhesiolysis and PRP treatment, with LPO-EMT <6 mm. Exclusion criteria included untreated genital tract infection, uncorrected uterine malformation, endometrial hyperplasia or malignancy, uncontrolled systemic disease, coagulation disorders, allergy to G-CSF, and contraindications to pregnancy.

### 2.3 PBPLC treatment regimen

The treatment regimen was *a priori* designed as a dual-axis intervention rather than as a surface intrauterine infusion of conventional platelet-rich plasma. This design reflected the hypothesis that, in women with severe refractory disease, the pathological process is unlikely to be confined to the endometrial surface and is likely to involve the basalis, the endometrial–myometrial junction, and subendometrial tissue. The regimen therefore combined two pre-specified components delivered within a single treatment cycle. The first was a cellular-enrichment component: G-CSF was used to expand the circulating leukocyte and progenitor-cell pool of the patient prior to peripheral blood collection, so that the resulting product would contain not only platelets but also a leukocyte fraction and a small population of bone marrow–derived progenitor cells. Previous studies have suggested that G-CSF–mobilised progenitor cells may migrate or home toward sites of tissue injury in response to local injury-derived chemotactic signals (14, 15). To preserve this enriched cellular cargo in the final product, the kit selected for processing was deliberately not a conventional leukocyte-poor PRP kit but a triple-chamber, customisable platelet–leukocyte concentrate kit (Tricell) routinely used in orthopaedic regenerative practice and reported in independent characterisation work to produce a CD34+ cell-containing platelet-rich product (16). The second was a deep-local delivery component: rather than instillation of the resulting concentrate onto the endometrial surface, the PBPLC was delivered by hysteroscopic injection into multiple sites at the endometrial–myometrial junction. Together, these two components were intended to expose the deeper compartments of the uterus to a more cellularly enriched regenerative product than is achievable with conventional surface PRP infusion. Throughout this manuscript, this combined design is therefore referred to as a cellularly enriched, dual local–systemic regenerative regimen; the term “systemic” refers specifically to the use of G-CSF to mobilise the patient’s own cellular pool into the product and not to the systemic delivery of progenitor cells back to the patient.

All eligible patients were assigned to receive a single course of G-CSF mobilization followed by hysteroscopic intrauterine administration of autologous PBPLC. Lenograstim (glycosylated rhG-CSF; Neutrogin^®^, Chugai Pharmaceutical, Tokyo, Japan) was administered subcutaneously at 10 µg/kg as a single daily dose for 3 consecutive days, with peripheral blood collection on day 4. The 10 µg/kg/day dose was selected because dose-finding studies in healthy subjects have shown that this is the minimum dose at which the great majority of healthy donors reach peripheral CD34+ cell concentrations sufficient for stem-cell collection (17, 18). The number of dosing days was deliberately shortened from the 5-day schedule used in apheresis-based mobilisation of allogeneic stem-cell donors to a 3-day schedule, because in this clinical population a longer mobilisation period (≥4–5 days) consistently produced severe systemic symptoms (myalgia and generalised bone pain) and was poorly tolerated. With this shortened schedule, day 4 was retained as the collection day, in keeping with the day 4 or day 5 collection windows used in established peripheral blood progenitor-cell programmes (19); we acknowledge, however, that day 4 collection after a 3-day mobilisation falls earlier than the day 5–6 peak reported in 5-day schedules (18), and that the resulting peripheral CD34+ concentrations would therefore be expected to lie in the lower part of, rather than at the peak of, the published mobilisation range. Exploratory flow-cytometric characterization was performed by CHA Biotech Co., Ltd. using one representative patient sample following G-CSF priming and buffy coat isolation. The panel included CD34 and CD45 to characterize hematopoietic progenitor-associated and leukocyte populations, respectively, and CD73 and CD90 to explore mesenchymal-associated marker expression. Accordingly, these flow-cytometric data were used to provide preliminary immunophenotypic characterization of the PBPLC product.

90 mL of peripheral venous blood was aseptically collected into ACD-A–coated syringes and processed using Tricell PRP kits (Blue Meditech Co., Ltd. Seoul, Korea). The samples underwent two-step centrifugation (first spin: 3000 rpm for 5 min using three sterile 30-mL kits), after which blood separated into red blood cells, buffy coat, and plasma. The plasma and buffy coat fractions were further processed by a second centrifugation. After the second spin, the platelet- and leukocyte-rich concentrate in the upper collection chamber and the buffy coat fraction were harvested. Only these fractions were collected, yielding approximately 5 mL of autologous PBPLC, which was administered intrauterinely within 1 hour of processing. All participants had completed prior hysteroscopic adhesiolysis as part of standard care before study entry, and were enrolled only after they remained refractory despite this and other treatments. At V-0, hysteroscopy was performed for cavity assessment, AFS scoring, and PBPLC delivery, with no adjunctive therapy provided thereafter. Minor residual fibrotic strands encountered at the time of PBPLC administration were lysed only when they impeded access for injection; no formal repeat adhesiolysis aimed at cavity restoration was undertaken. The intervention under evaluation was therefore PBPLC administration delivered into an anatomically prepared but biologically refractory cavity, rather than a combined adhesiolysis-plus-PBPLC procedure. Using a cystoscopic needle, approximately 3 mL of PBPLC was injected into 5–6 subendometrial sites at the endometrial–myometrial junction (0.5 mL per site), including the central fundal region, with the remaining injection sites distributed circumferentially in the bilateral cornual and adjacent lateral uterine wall regions, approximately 1 cm from each tubal ostium. The remaining suspension was evenly distributed over the endometrial surface.

### 2.4 Safety assessment

AEs were assessed by the investigator from G-CSF mobilisation through the follow-up period. Clinical monitoring included assessment of symptoms and signs potentially related to treatment, particularly bone pain, fever, hypersensitivity reactions, bleeding events, uterine complications, embolic events, and tumour development. Laboratory safety monitoring included serial complete blood count parameters, including white blood cell count, platelet count, haemoglobin, and red blood cell count, at predefined study visits to identify haematological changes after treatment. AEs were collected and managed in accordance with Korean Good Clinical Practice (KGCP), and their severity (including laboratory abnormalities) was graded using the Common Terminology Criteria for Adverse Events (version 5.0), with Grade 3 or higher designated as serious AEs. A participant could be removed from the study for the following reasons: unresolvable grade 3–4 drug-related AEs or other serious medical conditions unrelated to the study drug, non-compliance and unforeseeable conditions that substantially impede study data collection, or participant’s willingness to withdraw from the study with informed consent.

### 2.5 Fertility treatment

After treatment, conception attempts were managed individually according to each patient’s reproductive plan and clinical condition. For patients undergoing embryo transfer (ET), endometrial preparation was tailored according to ovulatory status and prior treatment response, using either a natural/modified natural cycle or an artificial hormone-replacement cycle. In general, ET was considered when the uterine cavity had been satisfactorily restored and the late proliferative endometrium reached a clinically acceptable threshold, preferably with an endometrial thickness of ≥6 mm. Cycles with persistently thin endometrium (<6 mm) or clearly suboptimal morphology were individually reassessed, and transfer timing was deferred or the preparation strategy was modified at the discretion of the treating physician. One patient attempted conception by timed intercourse rather than ET.

### 2.6 Endpoints

The primary endpoint was the change in LPO-EMT from baseline (V-0) to 1 month after PBPLC treatment (V-1). Transvaginal ultrasonography was performed on menstrual cycle days 8–12 to measure LPO-EMT. Secondary endpoints included safety, changes in menstrual blood loss, hysteroscopic improvement and early pregnancy outcomes. Participants had a hospital visit from day 1 to 3, every 2 weeks to week 4, and then in month 2, 4, 6, 8, and 12. During the first 3 days, we collected blood samples for complete blood counts, evaluated AEs and concomitant medication use. At week 4, second-look hysteroscopy was performed. At each visit, physical examinations were performed, AEs were assessed, and menstrual blood loss was evaluated. Hysteroscopic improvement was defined as a reduction in adhesion severity at V-1, as assessed by the AFS scoring system, together with restoration of uterine cavity configuration (including improved cavity patency and visibility of anatomic landmarks such as the tubal ostia). Patients then proceeded to fertility treatment as clinically indicated. Early pregnancy outcomes were defined by ultrasound visualization of an intrauterine gestational sac and were followed for up to 1 year (V-2). The protocol was approved by the Institutional Review Board.

### 2.7 Statistical analysis

This pilot study was restricted to women with SRAS, a rare and highly selected population, recruitment was inherently limited. Therefore, a sample size calculation was not done. In settings with limited prior data, pilot studies commonly use small sample sizes in the range of approximately 10–12 participants (20, 21). We assumed that ten participants were considered acceptable for this single-arm pilot study, while recognising that the analyses were exploratory and not confirmatory trials.

Continuous variables were summarised as mean (SD) or median [interquartile range (IQR)], as appropriate, and categorical variables as number (%). Exact (Clopper-Pearson) 95% confidence intervals (CIs) were calculated for key proportions where appropriate. The primary efficacy endpoint was analyzed using the exact Wilcoxon signed-rank test. The treatment effect was summarised as the Hodges-Lehmann estimate of the paired difference with an exact 95% confidence interval (CI). Changes in hysteroscopic AFS score and continuous laboratory safety variables were analysed in the same way. Menstrual improvement, pregnancy outcomes, and safety assessments were analysed descriptively. As this exploratory pilot study included multiple secondary endpoints, no adjustment for multiplicity was applied and p values are reported descriptively. All tests were two-sided, and nominal p<0.05 was considered statistically significant. Analyses were performed using R (version 4.2.2) and SPSS (version 26).

## 3 Results

**Figure 1:**
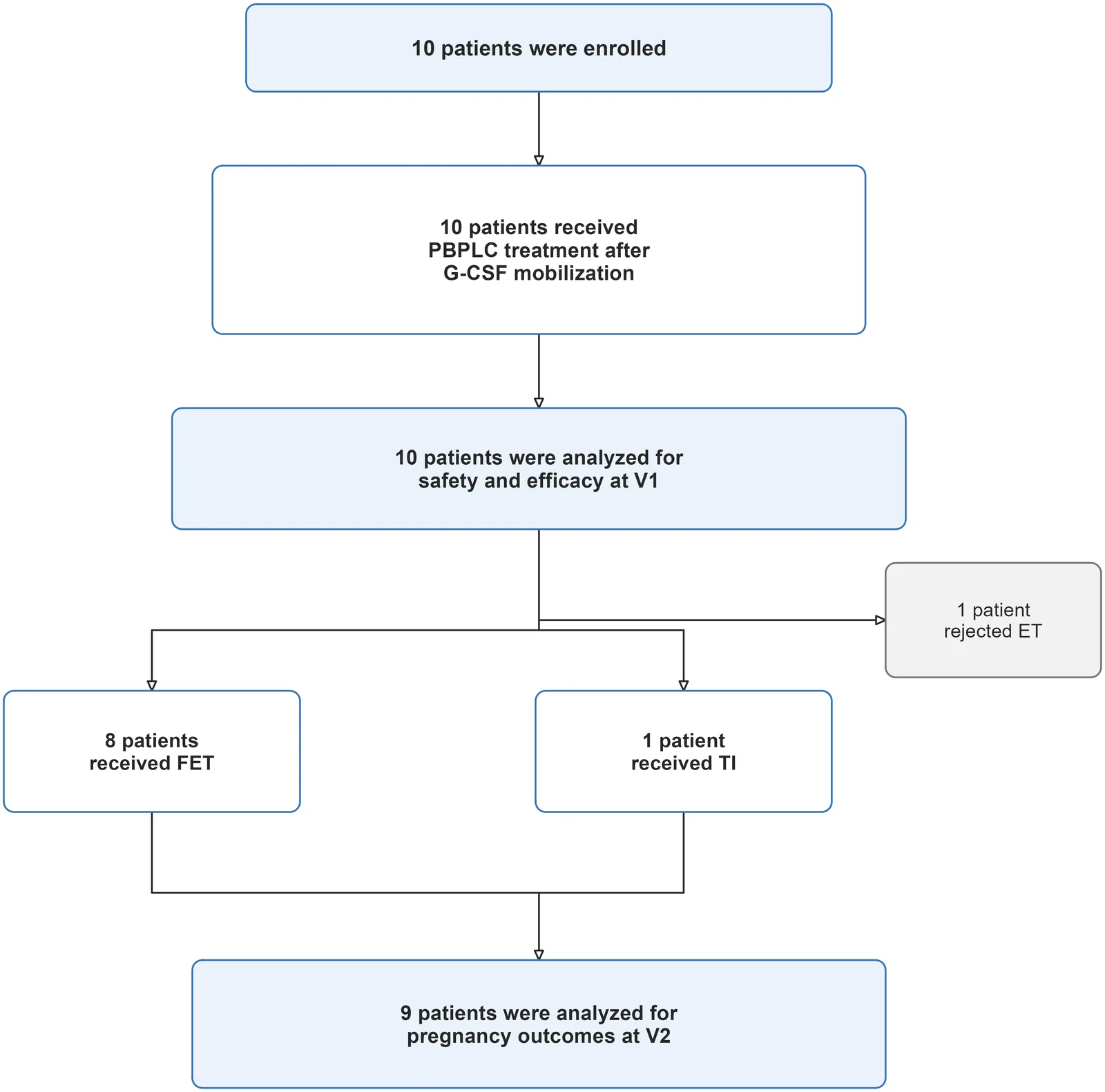
Flow chart of the study.

A total of 10 infertile women with SRAS were screened and enrolled into the study. All ten participants received a single course of PBPLC. Nine patients proceeded to conception attempts, comprising frozen-thawed embryo transfer (FET) in 8 patients and timed intercourse (TI) in 1 patient (**Figure 1**). As of the data cut-off date (1 July 2022), the median follow-up time for activity analyses was 32.6 months (IQR 21.7-34.1).

Baseline characteristics are summarized in **Table 1**. Participants’ ages ranged from 34 years to 42 years (mean 36.9, SD2.8), and BMI ranged from 20.8 to 25.4 kg/m^2^ (mean 22.3, SD1.4). The duration of infertility ranged from 3 years to 12 years (mean 6.3, SD2.7). The aetiology of severe refractory Asherman syndrome in this cohort, where identifiable, comprised dilatation and evacuation (D&E) in five patients (50%; including three with repeated procedures performed two, four, and five times, respectively), uterine artery embolisation (UAE) in two patients (20%), pelvic tuberculosis in one patient (10%), and pelvic radiotherapy for colorectal cancer in one patient (10%); one patient had a history of both D&E and UAE, and two patients had no clearly identifiable cause of endometrial injury. Importantly, every identifiable aetiology in this cohort — repeated D&E, UAE, tuberculous endometritis, and pelvic radiotherapy — may be associated with injury extending beyond the endometrial basalis to involve the endometrial-myometrial junction, the subendometrial vasculature, and the myometrium itself. The cohort therefore consists exclusively of women whose underlying disease is anatomically and biologically may not confined to the endometrial surface, which is directly relevant to interpretation of the reproductive outcomes reported below. Patients had undergone the number of previous hysteroscopies ranged from 1 to 6 years [median 2.0, IQR (1.0, 4.0)] and IVF attempts times ranged from 1 to 12 years [median 3.0, IQR (1.8, 5.5)].

**Table 1:** Characteristics of participants (n=10).

Characteristic	Patients (n = 10)
Age, years, mean (± SD)	36.9 (± 2.8)
BMI (kg/m^2^), mean (± SD)	22.3 (± 1.4)
Duration of infertility, years, mean (± SD)	6.3 (± 2.7)
FSH, IU/L, mean (± SD)	7.6 (± 1.8)
AMH, pg/mL, mean (± SD)	3.0 (± 2.3)
LH, IU/L, mean (± SD)	4.7 (± 2.9)
WBC, µL, mean (± SD)	6208 (± 2874.5)
Hysteroscopy times, median (IQR)	2.0 (1.0, 4.0)
IVF times, median (IQR)	3.0 (1.8, 5.5)

Data are presented as mean ± SD or median (IQR), as appropriate. All characteristics were assessed at baseline before PBPLC treatment. BMI, body mass index; FSH, follicle-stimulating hormone; AMH, anti-Mullerian hormone; LH, luteinizing hormone; WBC, white blood cell count; IQR, interquartile range; SD, standard deviation; IVF, *in vitro* fertilization.

**Figure 2:**
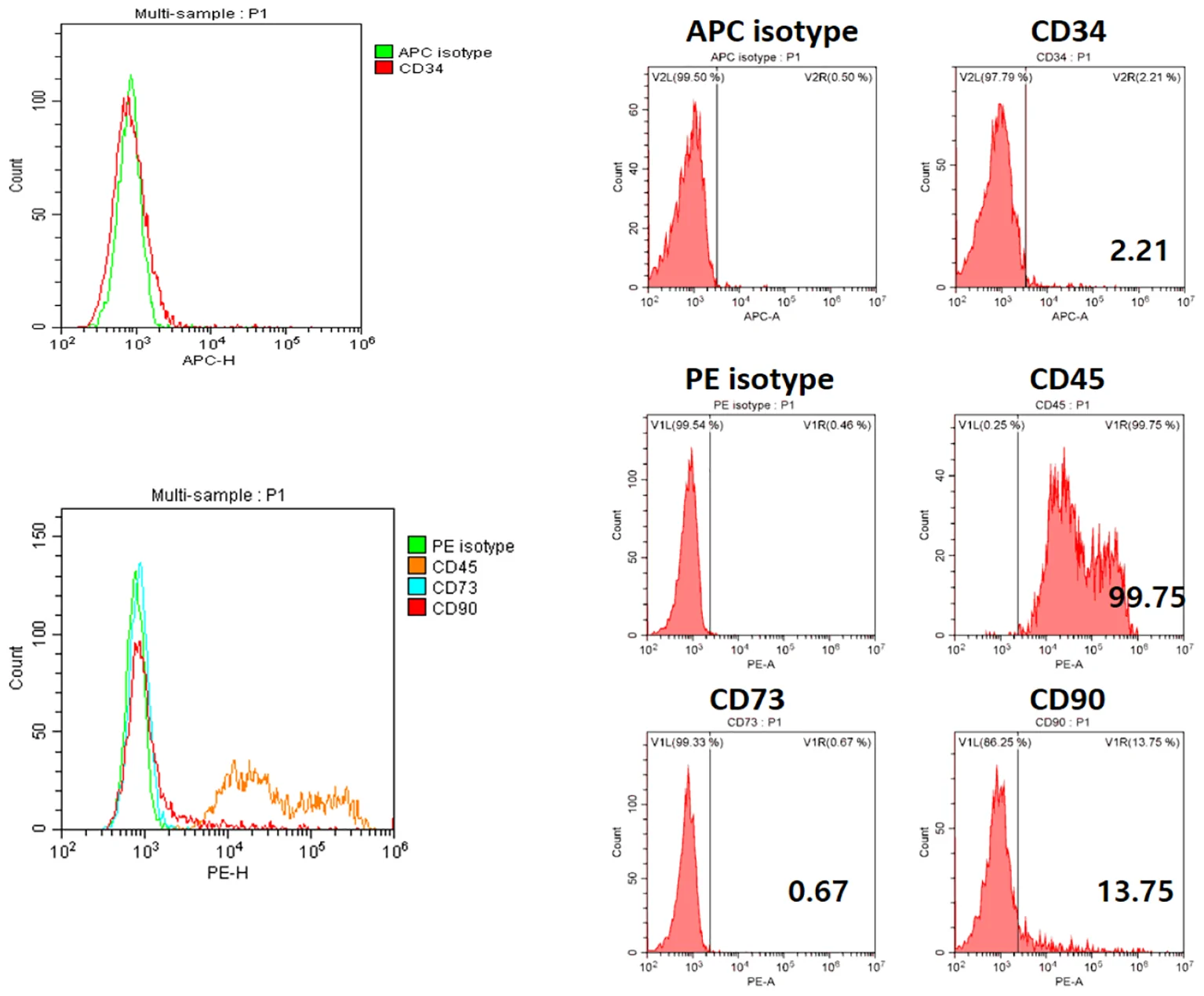
The analysis of CD markers (CD34, CD45, CD73, CD90) from peripheral blood using flow cytometry.

Mobilization was supported by post–G-CSF leukocytosis in all patients and flow-cytometric characterization of leukocyte populations (**Figure 2**). White blood cell (WBC) counts increased to 35, 095 ± 8991/µL (range, 15, 650–47, 820/µL) within 72 h after G-CSF administration (p < 0.001). Peripheral blood was predominantly composed of CD45^+^ leukocytes (99.75%), with a small CD34^+^ population (2.21%) consistent with mobilized hematopoietic progenitor cells. Mesenchymal-associated markers were infrequent (CD73^+^ 0.67%) and CD90^+^ was detected in 13.75% of analyzed cells. Isotype controls showed >99% negative signals, supporting assay specificity. Collectively, these findings indicate that G-CSF mobilisation predominantly induced circulating leukocytes and a minor population of hematopoietic progenitor cells.

### 3.1 Primary endpoints

**Figure 3:**
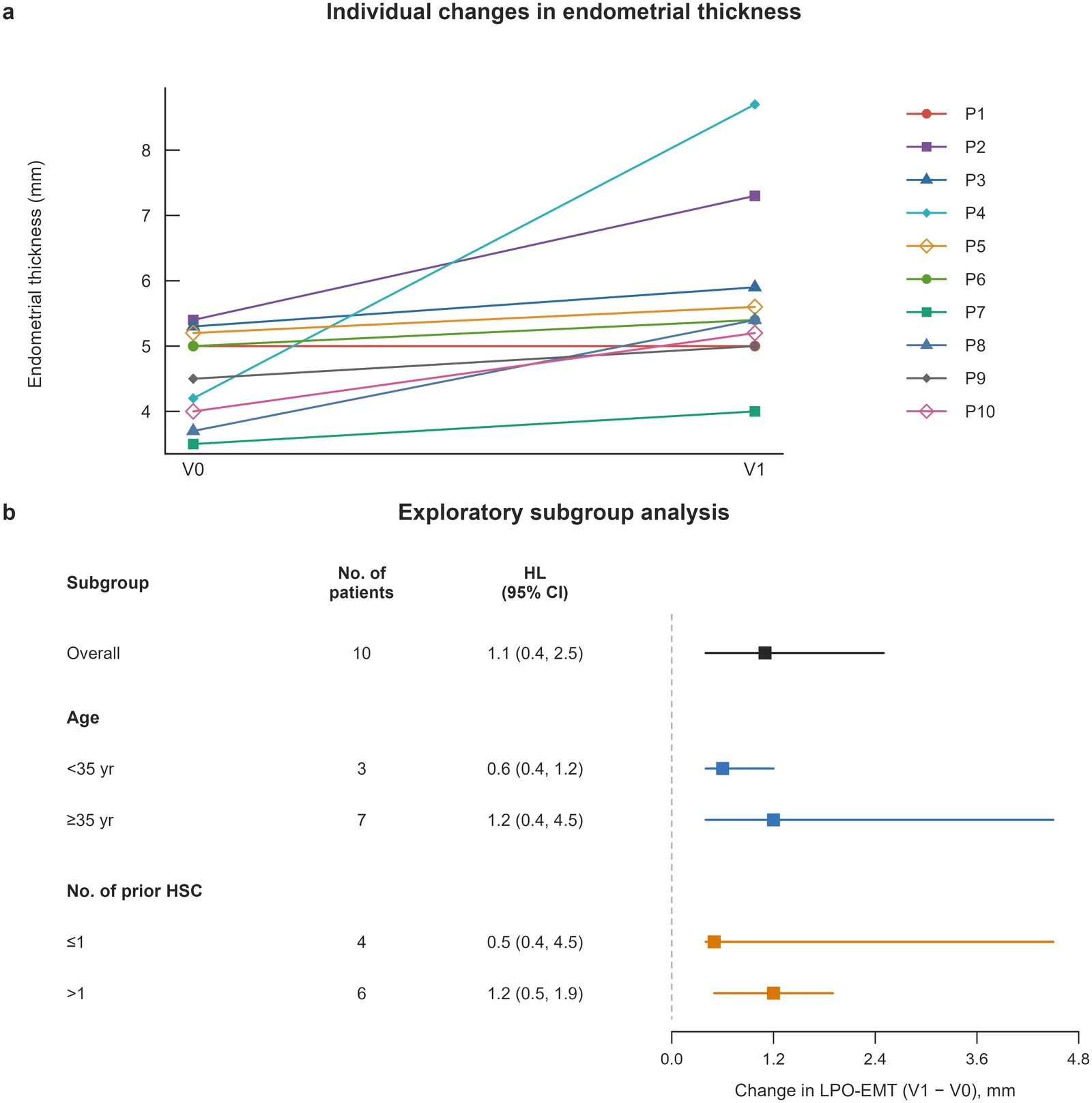
Overall and Subgroup Changes in LPO-EMT After Treatment. **(a)** Change in LPO-EMT across all patients. **(b)** Exploratory subgroup analyses by age and number of prior HSC procedures showed a generally positive direction of LPO-EMT change across subgroups.

Second-look hysteroscopy was performed at V-1 following hysteroscopic intrauterine administration of autologous PBPLC. LPO-EMT increased from 4.6 (SD0.7) to 5.8 (SD1.3). The HL estimate of the paired difference was 1.08 mm (95% CI 0.45 to 2.50; p=0.0039). One participant showed no change in LPO-EMT, whereas the remaining nine participants demonstrated an increase between V-0 and V-1 (**Figure 3a**). The change in LPO-EMT benefits appeared generally consistent across all the prespecified subgroups, including age and the number of previous hysteroscopic (HSC) procedures (**Figure 3b**).

### 3.2 Secondary endpoints

**Figure 4:**
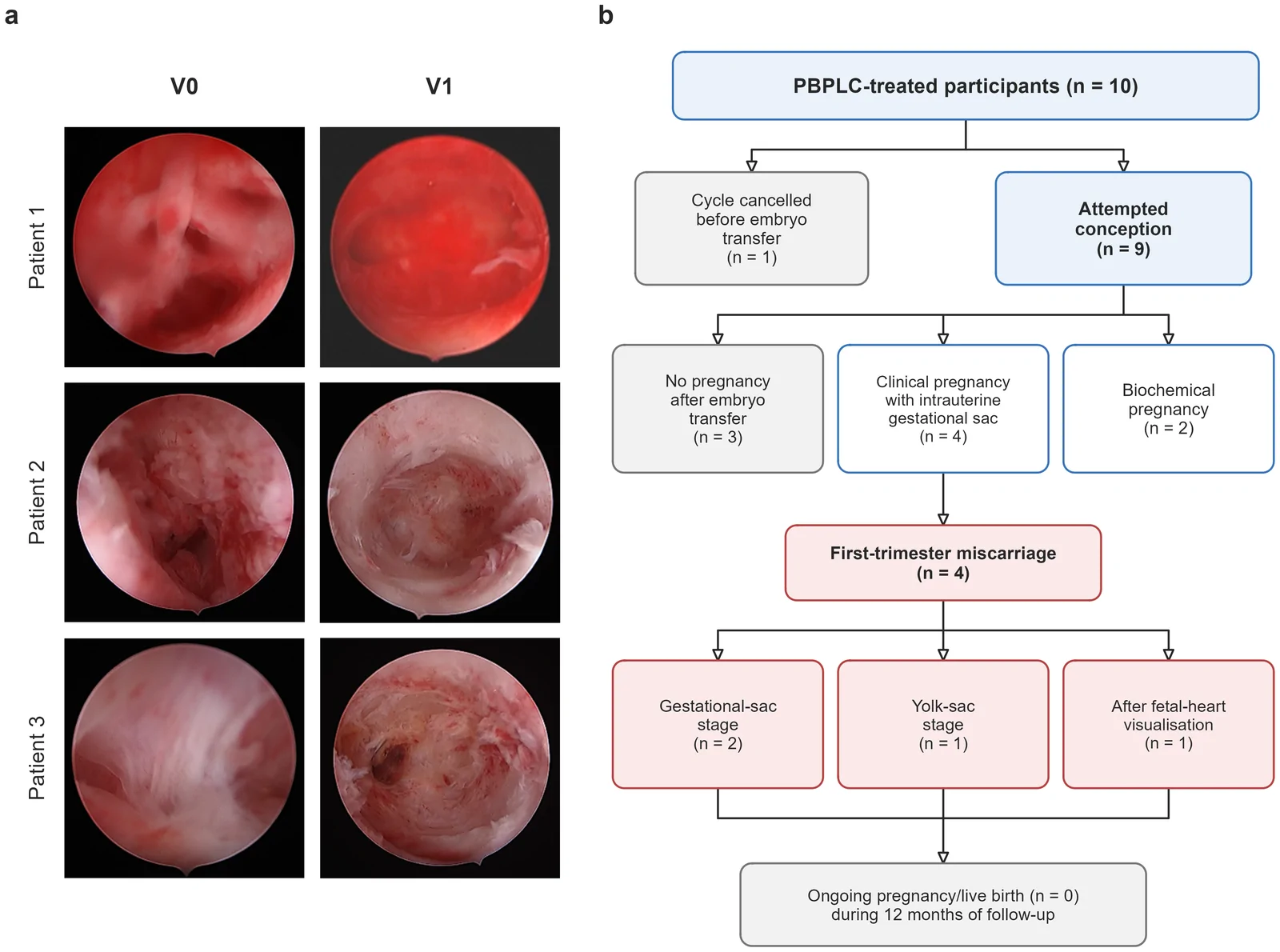
Hysteroscopic and reproductive outcomes after PBPLC. **(a)** Representative improvement in uterine cavity morphology. **(b)** Reproductive outcome after PBPLC.

HSC evaluation demonstrated marked anatomic improvement, with restoration of uterine cavity configuration and visibility of the tubal ostia. At 4 weeks after PBPLC injection, the AFS score decreased from 10.8 (SD 1.0) to 4.5 (SD1.1). The Hodges-Lehmann estimate of the paired reduction was 5.8 points (95% CI 5.5 to 7.0; p=0.0020). Among the individual AFS components, uterine cavity involvement contributed the largest proportion of the overall reduction (44.4%), followed closely by adhesion type (36.5%), whereas menstrual-status changes accounted for 19.1% (**Supplementary Table 1**). In addition, all participants reported subjective improvement in menstrual flow. Representative hysteroscopic images before and after treatment are shown in **Figure 4a**.

Detailed patient-level endometrial and embryological characteristics at the time of ET for the eight patients who underwent FET are presented in **Supplementary Table 2**. At the time of ET, the median endometrial thickness was 6.0 mm (range, 5.0–8.5 mm). A triple-line endometrial pattern was observed in four patients, whereas the remaining four had a non-triple-line pattern; no intracavitary fluid was detected in any patient. Reproductive outcomes were evaluated in nine patients, comprising eight who underwent FET and one who conceived naturally. Pregnancy was documented in six of these nine patients (66.7%; exact 95% CI 29.9 to 92.5), including 4 clinical pregnancies defined by the presence of a gestational sac (44.4%; exact 95% CI 13.7 to 78.8), and 2 biochemical pregnancies (22.2%; 95% CI 2.8 to 60.0) (**Figure 4b**).

### 3.3 Safety

The most common treatment-related AEs were leukocytosis and transient bone pain, each observed in 10/10 patients (100%). Bone pain developed within 3 days after G-CSF mobilization and resolved spontaneously without sequelae. Among the 10 participants, two (20.0%) developed mild thrombocytopenia (Grade 1) at V-1 without any bleeding-related symptoms or signs, including petechiae, purpura, gingival bleeding, or epistaxis. Hemoglobin and red blood cell count showed no significant change (**Table 2**). No grade ≥3 AEs were observed. All AEs were attributed to the intervention and resolved by V-2, with no lasting effects. Importantly, no participants experienced PBPLC-related fever, anaphylactic reactions, uterine rupture, severe hemorrhage, embolism, or the development of tumors throughout the follow-up period.

**Table 2:** The parameters related to the safety evaluation in patients pre- and post- PBPLC at V-1.

Test items (normal value range)	Pre-PBPLC	Post-PBPLC	HL estimate, (95% CI)	p value
Red blood cell count (3.7-5.2 x10^6^/μL)	4.52 (0.36)	4.42 (0.31)	-0.1 (-0.3 to 0.0)	0.262
Hemoglobin (11–16 g/dL)	13.62 (1.02)	13.26 (1.03)	-0.4 (-0.9 to 0.1)	0.143
Platelet count (150–380 x10^3^/μL)	252.20 (61.84)	205.90 (66.10)	-38.5 (-93.5 to -13.0)	0.010
White blood cell count (4–10 x 10^3^/μL)	6.21 (2.87)	35.09 (8.99)	29.3 (22.1 to 33.3)	0.001

Data are mean (SD); P values were calculated using the exact Wilcoxon signed-rank test.

## 4 Discussion

In this prospective, single-arm pilot study, we evaluated the feasibility, safety and preliminary activity of G-CSF-mobilised autologous PBPLC injected into the endometrial–myometrial junction in women with SRAS. To our knowledge, this is the first clinical study to evaluate the efficacy and safety of PBPLC as an intrauterine regenerative strategy in this difficult-to-treat population, with promoting endometrial growth and improving uterine-cavity integrity.

Among the patients with thin endometrium, PRP was associated with greater increases in EMT and higher embryo-implantation rates than conventional hormone-replacement therapy. In our prior clinical experience in refractory thin endometrium (EMT), PRP was also associated with improvements in EMT (8). However, the SRAS uterus differs from refractory thin endometrium in that the dominant pathological process is not simply impaired endometrial growth but a chronic, fibrosing, and pro-inflammatory injury that involves the basalis, the underlying stroma, and adjacent uterine compartments. In this setting, a regenerative product that combines platelet-derived growth factors with a G-CSF-mobilised leukocyte fraction may be better suited to support resolution of chronic injury and tissue remodelling than to deliver further growth-factor stimulation alone, providing a biological rationale for the PBPLC strategy evaluated here.

For patients with SRAS, current first-line treatments consists of hysteroscopic adhesiolysis under direct visualization, together with adjuvant postoperative measures (22). Commonly used approaches include intrauterine devices (reported 1-month posttreatment recurrence, 30%), intrauterine balloons (35%), estrogen therapy (26.7%), and hyaluronic-acid–based barriers (31.1%) (23). In this context, PRP has emerged as an alternative treatment option. In a randomized, controlled trial (RCT) involving patients with moderate-to-severe IUA (n=123), PRP was associated with a slightly lower re-adhesion rate than control (20.6% vs. 30.0%) (24). A systematic review and meta-analysis restricted to RCTs (5 trials; 329 patients) reported that PRP reduced severe recurrence from 23.4% to 7.6% (25). A recent retrospective cohort (299 patients with moderate-to-severe IUA, all receiving PRP) suggested that subendometrial injection was associated with higher menstrual improvement than intrauterine infusion (77.0% vs. 52.9%) and with signals of improved clinical pregnancy after multivariable adjustment and propensity-score matching (26). In IUA mice models, PRP has been associated with reduced collagen deposition and downregulation of fibrosis-related markers, including COL1A1, TGF-β1, TIMP1, α-SMA, and fibronectin, together with enhanced angiogenic and tissue-repair signalling (27, 28). PRP may also potentiate the functional activity of reparative cell populations by promoting metabolic fitness and enhancing pro-angiogenic paracrine secretion (29).Against this background, we designed an approach in which PRP was co-infused with G-CSF–mobilized peripheral blood mononuclear fraction (PBMC-rich buffy coat) and administered hysteroscopically. Post-G-CSF leukocytosis and flow-cytometric profiling of leukocyte populations were consistent with effective peripheral mobilization; however, the product is not a purified stem-cell preparation. Rather, it consisted of a PBMC-rich fraction containing predominantly CD45^+^ leukocytes with relatively rare CD34^+^ cells and low levels of mesenchymal markers. This cellular profile is in line with previous evidence showing that G-CSF is a prototypical mobilizing agent capable of inducing the egress of bone marrow–resident hematopoietic stem/progenitor-cell–associated populations into the peripheral circulation, partly through remodeling of the SDF-1/CXCR4 axis and the bone marrow niche (14, 30). Moreover, mobilized bone marrow–derived progenitor populations have been implicated in regenerative trafficking toward injured or ischemic tissues, where they may contribute to repair through angiogenic, immunomodulatory, and paracrine mechanisms (31). Although tissue homing was not directly demonstrated in the present study, earlier human studies have shown that bone marrow–derived cells can contribute to endometrial cell populations (32), and recent clinical studies using autologous CD133^+^ bone marrow–derived stem cells have reported improved endometrial thickness, vascularity, menstrual function, and reproductive outcomes in patients with refractory Asherman syndrome or endometrial atrophy (33, 34). Therefore, we hypothesize that G-CSF–induced systemic mobilization may have contributed to regenerative trafficking toward the injured endometrial microenvironment. In addition, direct injection of the PBPLC product into the endometrial–myometrial junction may have further enhanced local cell–microenvironment interactions at the site of endometrial injury. This “whole-fraction” strategy may trade cellular precision for feasibility, scalability, and accessibility—an important consideration for a condition that often requires repeated procedures and long follow-up.

In our study, the primary objective of demonstrating feasibility was met. A short, 3-day G-CSF mobilization protocol was sufficient to achieve the expected hematologic mobilization profile and enabled timely bedside preparation and hysteroscopic delivery of the PBPLC product. We observed evidence of endometrial recovery, including an increase in EMT and improvements on hysteroscopy, with reductions in adhesion severity and restoration of uterine-cavity architecture. It is important to note that, in this trial, PBPLC was administered at hysteroscopy without concurrent formal adhesiolysis; the observed reduction in AFS score and the restoration of cavity configuration therefore reflect activity of the regenerative product itself rather than the mechanical effect of repeated bands lysis. These findings support the concept that, even in “end-stage” fibrotic uteri, a locally delivered regenerative strategy may help re-establish a more functional endometrial environment.

The reproductive outcomes provided the most informative — and, in our view, the most clinically instructive — signal of this trial. Pregnancies occurred in six of nine evaluable women (66.7%) and four reached clinical pregnancy with a documented intrauterine gestational sac (44.4%). Although conception occurred in several participants, all clinical pregnancy ended in first-trimester loss; no ongoing pregnancy or live birth was achieved. Implantation, in other words, was attainable, but sustained placentation was not, suggesting that improvements in endometrial thickness and uterine-cavity morphology may not fully reflect reproductive competence. The apparent dissociation between post-treatment improvements in uterine cavity anatomy and endometrial thickness and the absence of ongoing pregnancy should be interpreted cautiously. These structural improvements do not necessarily indicate complete recovery of functional endometrial receptivity, nor can embryo-related factors be excluded, particularly given the small cohort, heterogeneity in embryo developmental stage and grade, and limited availability of PGT-A data.Persistent dysfunction involving deeper endometrial or uterine compartments—including stromal decidualization, junctional-zone integrity, spiral-artery and subendometrial vascular remodeling, and the local immune microenvironment—could hypothetically have contributed to the first-trimester pregnancy losses. However, these mechanisms were not directly evaluated, and their contribution cannot be determined from the present study.

Taken together with the anatomical and endometrial findings, these reproductive results invite a reframing of SRAS itself. The conventional model treats SRAS as an endometrial disease — a damaged or denuded basalis sitting within an otherwise normal uterus — and the therapeutic logic that follows is to thicken the endometrium and reopen the cavity. Our data suggest that this model is incomplete for end-stage disease. PBPLC delivered was sufficient to improve cavity architecture and endometrial thickness; yet this was not sufficient for any pregnancy to progress, even though the regimen had been deliberately designed as a dual-axis intervention combining systemic G-CSF mobilisation with deep subendometrial injection at the endometrial–myometrial junction. We therefore propose that severe refractory Asherman syndrome be conceptualised not as an isolated endometrial defect, but as a whole-uterus disorder of chronic inflammation and exhausted regeneration, in which the basalis, junctional zone, subendometrial vasculature, and myometrial stroma have all been remodelled by repeated injury and persistent pro-fibrotic signalling. Recent single-cell transcriptomic data from Asherman uteri are consistent with this view, demonstrating altered stromal and vascular niches that extend well beyond the surface epithelium (6). The aetiological composition of our own cohort is also consistent with this framing: every identifiable aetiology — repeated dilatation and evacuation, uterine artery embolisation, pelvic tuberculosis, and pelvic radiotherapy — characteristically inflicts injury extending beyond the endometrial basalis into the junctional zone, subendometrial vasculature, and myometrium, rather than being confined to the endometrial surface. Within such a framework, the four first-trimester losses observed in this cohort are not a paradox of an otherwise positive trial, but the expected behaviour of pregnancies implanting in a uterus whose deeper regenerative compartments remain refractory. The therapeutic implication is sobering: even when intrauterine regenerative therapy is augmented by systemic G-CSF mobilisation and delivered by deep subendometrial injection at the endometrial–myometrial junction — as in the present trial — it may remain insufficient to convert implantation into ongoing pregnancy in end-stage disease. What is required is therefore unlikely to be further intensification of regenerative input within the same local–systemic framework, but fundamentally different therapeutic strategies that engage aspects of uterine biology — junctional-zone integrity, deep stromal decidualisation, and vascular remodelling — that current intrauterine cell-based approaches do not reliably address.

Safety findings were reassuring. No serious adverse events were observed. As expected, leukocytosis occurred during G-CSF mobilization, and transient bone pain was common and self-limited. Apart from leukocytosis, hematologic indices showed no clinically meaningful deterioration. These data support the clinical tolerability of a short G-CSF mobilization course and the intrauterine administration procedure in this setting.

This study has several limitations. First, this was a small, single-centre, single-arm pilot study without a control group and was intentionally restricted to women with SRAS. Although this restriction enhances the clinical relevance of the findings to this difficult-to-treat population, the study design limits causal inference and the generalisability of the findings to broader patient populations. Second, menstrual improvement was based on subjective patient report rather than a validated quantitative measure. Third, the PBPLC product was characterised primarily by surface immunophenotype, with limited flow-cytometric data and without quantification of absolute cell doses, viability, or growth-factor content; mechanistic and dose-response inferences should therefore be made with caution. Fourth, the proposed reframing of SRAS as a whole-uterus disorder is supported here by the discordance between anatomical and pregnancy outcomes, but is not directly demonstrated by deep-compartment imaging or histology in this cohort and should be regarded as a hypothesis to be tested in dedicated studies. Finally, reproductive follow-up yielded only a small number of pregnancy events, all ending in early loss. These outcomes should be considered exploratory rather than evidence of clinical efficacy, and no conclusions can be drawn regarding ongoing pregnancy or live birth.

Despite these limitations, the study also has important strengths. It addresses a population with very limited therapeutic options, uses a prospective design, combines anatomical, endometrial, clinical and safety outcomes, and provides early human data on a hysteroscopically delivered autologous PBPLC approach in severe refractory disease. In that sense, the study should be viewed primarily as proof-of-concept.

## 5 Conclusions

**Figure 5:**
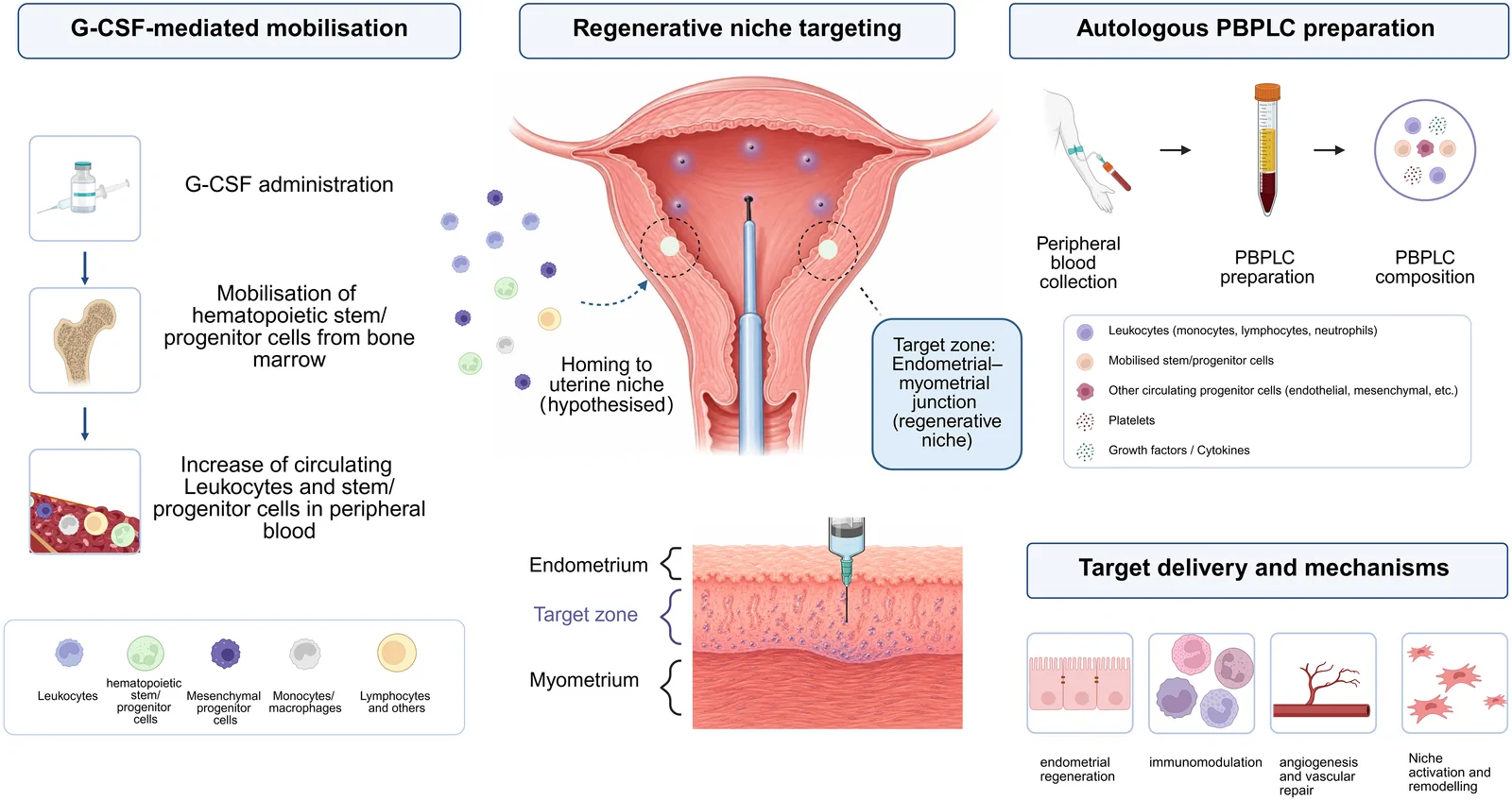
Proposed biological rationale and therapeutic strategy of G-CSF–mobilised autologous PBPLC therapy in SRAS.

In women with SRAS, a dual-axis regenerative regimen combining systemic G-CSF mobilisation with deep subendometrial injection of autologous PBPLC at the endometrial–myometrial junction, delivered at hysteroscopy without concurrent formal adhesiolysis, was feasible and well tolerated (**Figure 5**). The regimen was associated with anatomical restoration of the uterine cavity and an increase in endometrial thickness, and a substantial proportion of women conceived after treatment. However, every clinical pregnancy ended in first-trimester loss, with no ongoing pregnancy or live birth. These reproductive findings are exploratory and do not establish clinical efficacy. The underlying mechanisms, including whether abnormalities beyond cavity morphology and endometrial thickness contribute to pregnancy loss, require investigation in larger controlled studies incorporating embryo-related, vascular, histological, and endometrial functional assessments.

## Data Availability

The raw data supporting the conclusions of this article will be made available by the authors, without undue reservation.

## Supplementary material

Supplementary Table 1 Patient-level AFS component scores at V-0 and V-1.

Supplementary Table 2 Patient-level endometrial and embryological characteristics at the time of ET following PBPLC treatment.
