# Supplementary material for: First-in-human pilot trial of G-CSF–mobilised platelet–leukocyte concentrate for severe refractory Asherman syndrome
Source: Front Endocrinol (Lausanne). 2026 Aug 13;17:1923604. doi: 10.3389/fendo.2026.1923604 (PMC13518211; doi:10.3389/fendo.2026.1923604)
Supplement: Supplementary file 1 [file Table1.docx]

Supplementary Table S1. Patient-level AFS component scores at V-0 and V-1.

| Patient | V_0 | | | V_1 | | |
| --- | --- | --- | --- | --- | --- | --- |
|  | Cavity involvement | Adhesion type | Menstrual status | Cavity involvement | Adhesion type | Menstrual status |
| P1 | 4 | 4 | 4 | 2 | 2 | 2 |
| P2 | 4 | 4 | 2 | 1 | 2 | 2 |
| P3 | 4 | 4 | 2 | 1 | 1 | 2 |
| P4 | 4 | 4 | 2 | 1 | 2 | 0 |
| P5 | 4 | 4 | 4 | 1 | 2 | 2 |
| P6 | 4 | 4 | 2 | 1 | 2 | 0 |
| P7 | 4 | 4 | 2 | 1 | 2 | 2 |
| P8 | 4 | 4 | 4 | 1 | 1 | 2 |
| P9 | 4 | 4 | 2 | 1 | 1 | 2 |
| P10 | 4 | 4 | 4 | 2 | 2 | 2 |

| Patient | EMT at transfer (mm) | Endometrial pattern | Intracavitary fluid | Transfer type | No. of embryos transferred | Embryo developmental stage | Embryo grade | PGT-A | |
| --- | --- | --- | --- | --- | --- | --- | --- | --- | --- |
| P1 | 6.0 | Triple-line | No | FET | 3 | D4 | G1，G2 | - |  |
| P2 | 7.3 | Triple-line | No | FET | 3 | D4 | G2, G3 | - |  |
| P3 | 6.0 | Triple-line | No | FET | 1 | D5 | 6BB | Euploid |  |
| P4 | 8.5 | Non-triple-line | No | FET | 2 | D5 | 5BC | - |  |
| P5 | 6.0 | Non-triple-line | No | FET | 3 | D3 | G2, G3 | - |  |
| P6 | 5.0 | Non-triple-line | No | FET | 2 | D4 | G1 | - |  |
| P7 | 6.0 | Triple-line | No | FET | 2 | D4 | G1 | - |  |
| P9 | 6.0 | Non-triple-line | No | FET | 2 | D4 | G1 | - |  |

Supplementary Table S2. Patient-level endometrial and embryological characteristics at the time of ET following PBPLC treatment.

Note: – indicates not applicable. Transfer-related data were not applicable to P8, who conceived naturally, or P10, who declined assisted reproductive treatment.
